# Genetic similarities between tobacco use disorder and related comorbidities: an exploratory study

**DOI:** 10.1186/1471-2350-15-85

**Published:** 2014-07-24

**Authors:** Sylviane de Viron, Servaas A Morré, Herman Van Oyen, Angela Brand, Sander Ouburg

**Affiliations:** 1 Operational Direction Public Health and Surveillance, Scientific Institute of Public Health, J. Wytsmanstraat 14, 1050, Brussels, Belgium; 2 Institute for Public Health Genomics (IPHG), Department of Genetics and Cell Biology, Research Institute GROW (School for Oncology & Developmental Biology), Faculty of Health, Medicine & Life Sciences, Maastricht University, Maastricht, The Netherlands; 3 Laboratory of Immunogenetics, Department of Medical Microbiology and Infection Control, VU University Medical Center, Amsterdam, The Netherlands

**Keywords:** Cardiovascular disorders, Comorbidity, Genetics, Network, Psychiatric disorders, Public Health Genomics, Tobacco use disorder, Tobacco smoking

## Abstract

**Background:**

Tobacco use disorder (TUD), defined as the use of tobacco to the detriment of a person’s health or social functioning, is associated with various disorders. We hypothesized that mutual variation in genes may partly explain this link. The aims of this study were to make a non-exhaustive inventory of the disorders using (partially) the same genetic pathways as TUD, and to describe the genetic similarities between TUD and the selected disorders.

**Methods:**

We developed a 3 stage approach: (i) selection of genes influencing TUD using Gene2Mesh and Ingenuity Pathway Analysis (IPA), (ii) selection of disorders associated with the selected genes using IPA and (iii) genetic similarities between disorders associated with TUD using Jaccard distance and cluster analyses.

**Results:**

Fourteen disorders and thirty-two genes met our inclusion criteria. The Jaccard distance between pairs of disorders ranged from 0.00 (e.g. oesophageal cancer and malignant hypertension) to 0.45 (e.g. bladder cancer and addiction). A lower number in the Jaccard distance indicates a higher similarity between the two disorders. Two main clusters of genetically similar disorders were observed, one including coexisting disorders (e.g. addiction and alcoholism) and the other one with the side-effects of smoking (e.g. gastric cancer and malignant hypertension).

**Conclusions:**

This exploratory study partly explains the potential genetic components linking TUD to other disorders. Two principle clusters of disorders were observed (i) coexisting disorders of TUD and (ii) side-effects of TUD disorders. A further deepening of this observation in a real life study should allow strengthening this hypothesis.

## Background

Tobacco use disorder (TUD) is the greatest cause of preventable death in developed countries and is a well-known risk factor for many other disorders. It is defined by the Medical Subject Heading (MeSH) index [1], as “*Tobacco used to the detriment of a person’s health or social functioning. Tobacco dependence is included*”.

TUD is influenced by various environmental and genetic factors. Environmental factors encompass a broad range of cultural, social, and economic aspects. Genetic factors can be categorized into two main groups: Genes associated with the pathways related to nicotine metabolism, which indicates how fast someone is metabolizing nicotine into cotinine, and genes associated with the cascade theory of reward, which represents the amount of pleasure felt when smoking [2]. The most important genes influencing nicotine metabolism are cytochrome P450 *CYP2A6* and *CYP2B6*. Genes influencing the cascade theory of reward include the complex network of serotonin, opioid, gamma-aminobutyric acid (GABA), and dopamine [2].

Genes from these two groups have been found to influence traits and other disorders. For example, serotonin genes are associated with personality and psychiatric disorders such as depression [3,4]. Furthermore, TUD is related to a number of traits and disorders. For example, smoking increases the risk of several neoplasms [5] and a higher prevalence of smokers is observed in populations with schizophrenia [6]. Therefore, based on previous research, TUD is associated with a wide range of disorders but up to now, the mechanisms accounting for comorbidity of smoking are not well understood.

As proposed by Munafo *et al.* the relationship between, for example, smoking and depression may be (i) depression causes people to smoke (through self-medication of the symptoms), (ii) smoking causes an increased risk of depression (through alterations of neurotransmitter following chronic exposure to tobacco), (iii) bidirectional association (acute tobacco smoking reduces negative affect and chronic use increases it), (iv) caused by shared factors such as genetic factors, or (v) the combination of i-iii and iv. In the latter case, the relationship is not causal but due to pleiotropy that occurs when a single gene variant influences multiple phenotypic traits [7]. This raises questions about the relationship between TUD and other disorders: Is it due to causality, pleiotropy, or common pathways in systems medicine? Systems medicine is a field studying gene-environment interactions. Therefore systems medicine is not just the influence of genetics but also genomics such as epigenomics. Answering such questions is an important step to further improve the prevention and treatment of TUD comorbidities.

Given the number of disorders associated with TUD, combined with the importance of the genetic factors influencing TUD, the current study aims at exploring the genetic similarities between TUD and disorders genetically associated with TUD. Due to the wide array of disorders, this exploratory study only includes disorders that are highly associated with the genes influencing TUD. The aims of this study are (1) to make a non-exhaustive inventory of disorders using the same genetic pathway as TUD, (2) to describe the genetic similarities between TUD and the selected disorders.

## Methods

**Figure 1:**
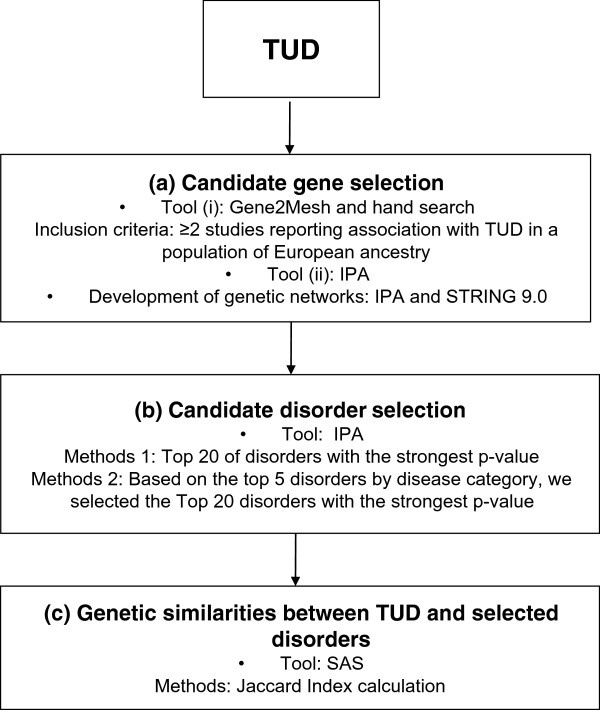
**Flow of the analyses.** Analysis proceeds from candidate gene selection, to candidate disorder selection, to genetic similarities between tobacco use disorder (TUD) and selected disorders. IPA, Ingenuity Pathway Analysis.

Figure 1 presents the analyses flow of the exploratory study described below.

### Candidate gene selection (Figure 1a)

To enhance the robustness of the candidate gene selection, two different tools were used: Gene2Mesh and Ingenuity Pathway Analysis (IPA).

Gene2MeSH screens all publications on PubMed for genes and MeSH terms and calculates the over-representation of each gene for each specific MeSH term [8]. In June 2012, a search was undertaken for genes over-represented in the literature for the following MeSH terms ‘*tobacco use disorder’*, ‘*nicotine’*, ‘*smoking*’ and ‘*smoking cessation*’ in human studies of English language. Genes were selected if at least two independent studies reported a significant association with TUD in a European population. Publications were excluded if the entire population had a specific disorder or trait other than nicotine dependence (e.g. alcohol addiction, psychiatric disorder or pregnancy).

IPA (Ingenuity Systems; https://www.ingenuity.com) allows the development of gene and gene-disease networks through different sources and databases including major NCBI databases (EntrezGene, RefSeq, and OMIM disease associations), microRNA-mRNA target databases, GWAS databases, and Kyoto Encyclopedia of Genes and Genomes (KEGG). Using IPA, all genes reported in the search using the term ‘*Tobacco use disorder*’ were selected in February 2013.

**Figure 2:**
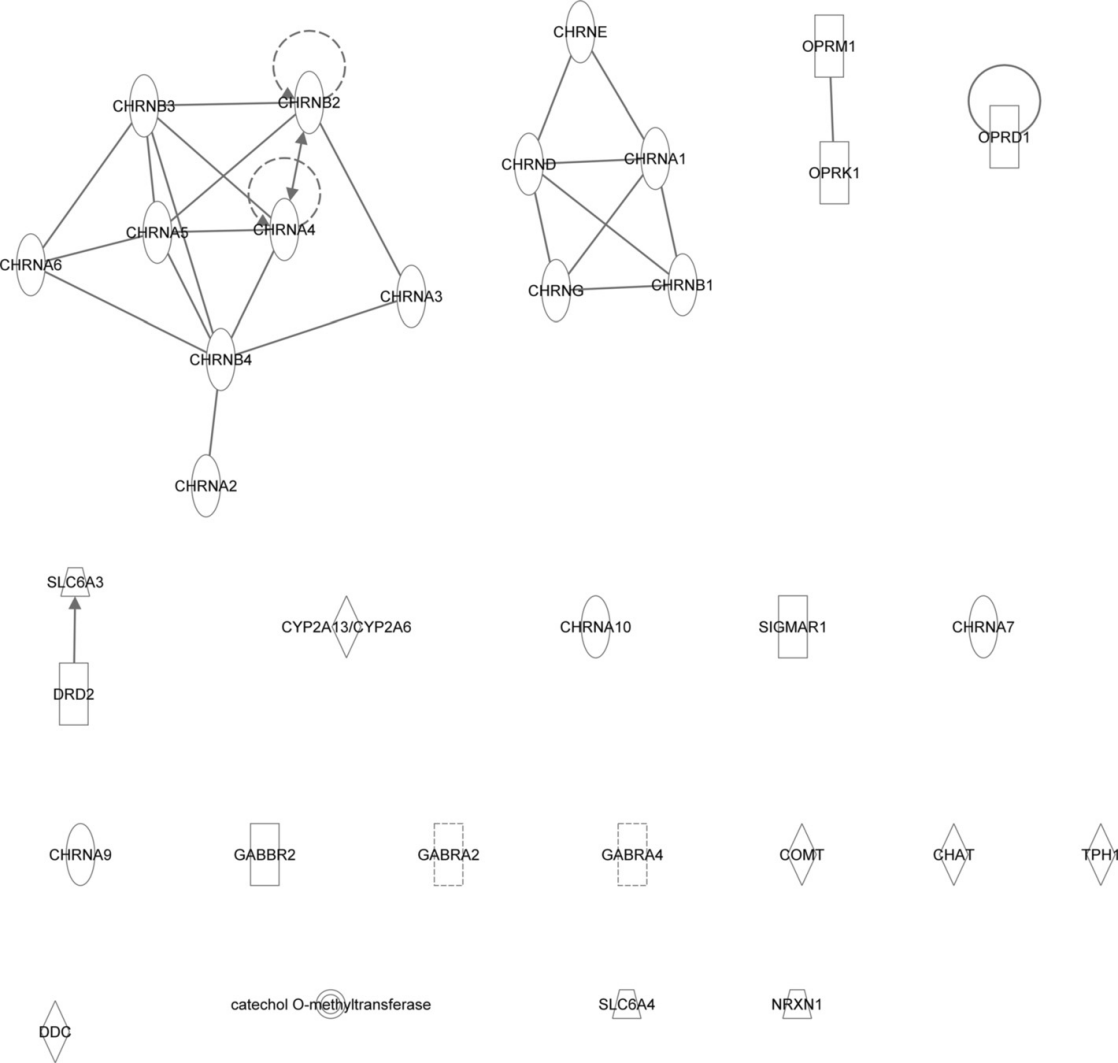
**Genetic network analyses using Ingenuity Pathway Analysis (IPA).** Genetic network obtained from Ingenuity Pathway Analysis. The different shapes of nodes represent the functional class of the gene product. Edges with dashed lines show indirect interaction, while a continuous line represents direct interactions.

**Figure 3:**
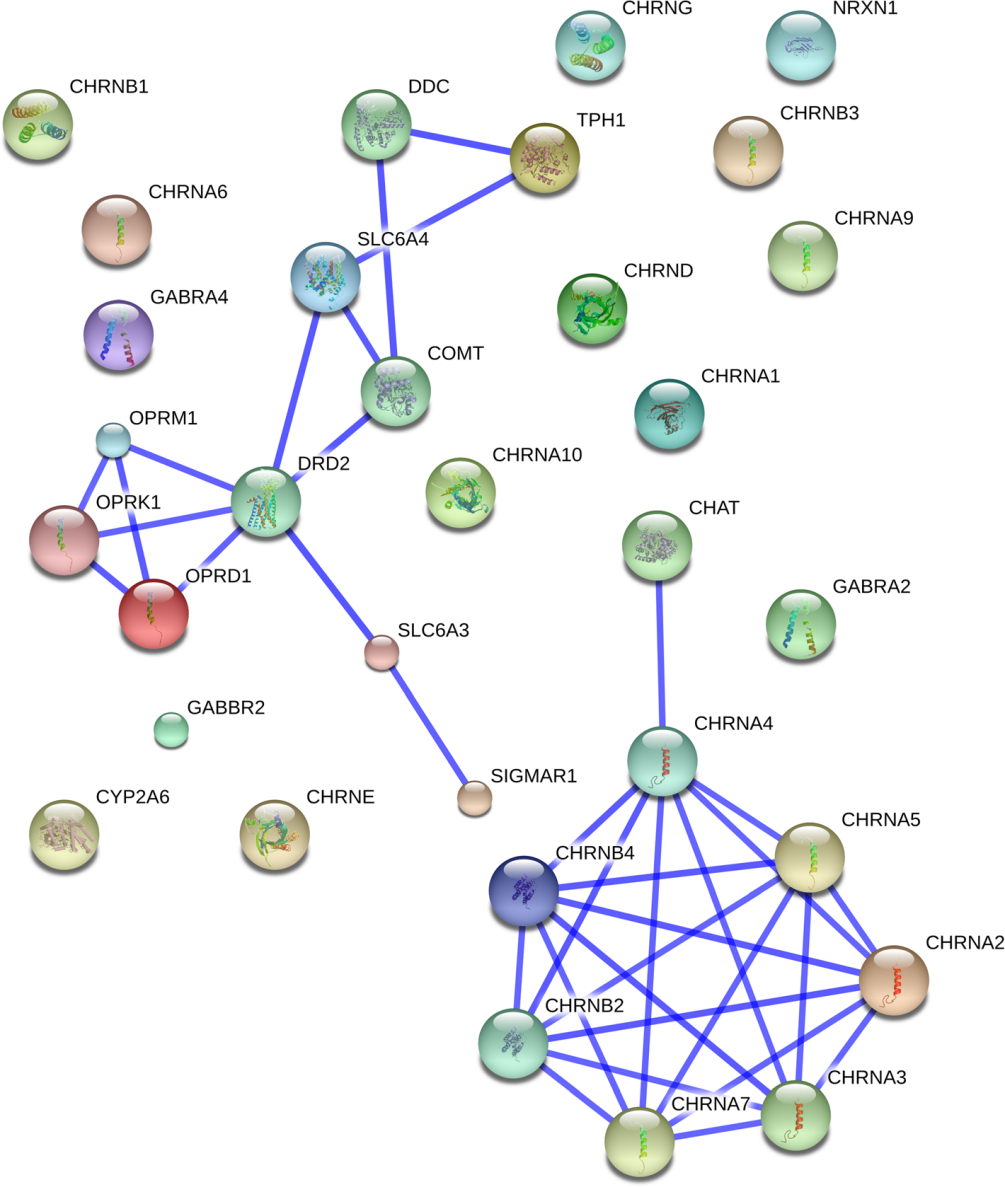
**Genetic network analyses using STRING.** Genetic network obtained from STRING network. The STRING network incorporates the interactions of the selected genes with the highest confidence level (level of 0.900). Stronger associations are represented by thicker lines.

Using IPA and STRING 9.0 (Search Tool for the Retrieval of Interacting Genes/Proteins), a network analysis was developed to gain insight into the functional relationships between the selected genes (Figure 2 and Figure 3) [9]. Genes that were identified by IPA as being related to TUD (Disease/function search) were selected for pathway analyses. Interactions between the genes were built using the following options: ‘Grow’ and ‘Path Explorer’ options filtered for direct interaction, experimentally observed/highly predicted confidence levels, the *Homo sapiens species*, and exclusion of chemicals and drugs. Interactions with only one supporting publication were manually excluded as “preliminary”. STRING is a web-tool providing gene-gene and protein-protein association scores based on automatic literature-mining searches. The list of selected genes was input in STRING and the minimum combined score was set to 0.900 (highest confidence). The model was built for *Homo sapiens*.

### Candidate disorder selection (Figure 1b)

Disorders were selected using IPA based on their association with the previously selected genes. As the number of associated disorders was expected to be high, two methods of selection were developed. Disorders were included only when retrieved using both methods. The first method selected the 20 disorders with the most statistically significant p-value indicating a strong association with the selected genes. In IPA, the p-value referred to a right-tailed Fisher’s exact tests calculating the likelihood that a set of genes was associated with a specific disorder. The second method was based on the category of disorders and was developed in two steps. The first step consisted in selecting the five disorders of each category with the strongest p-value. The second step consisted in selecting the 20 disorders with the strongest p-value. The combination of these two methods allowed the retrieval of broad categories of disorders having the most statistically significant association with TUD.

### Genetic similarities between TUD and selected disorders (Figure 1c)

A similarity matrix and a cluster analysis of disorders were developed based on genetic variations between disorders using the Jaccard distance [10]. The Jaccard distance measured the dissimilarity between pairs of disorders from a genetic point of view. Each gene was considered as a binary variable that was either ‘present’ or ‘absent’. Therefore, a lower number in the Jaccard distance indicated a higher similarity between the two disorders. Cluster analyses were undertaken using the centroid hierarchical method. The Jaccard distance was estimated using the DISTANCE procedure and a dendrogram was built using the CLUSTER and TREE procedures of SAS 9.2 (SAS Institute, Cary, NC).

## Results

### Candidate gene selection

Thirty-two genes were selected, 26 were retrieved from IPA and 12 from Gene2Mesh (Table 1). Seventeen of them were nicotinic receptors (*CHAT*, *CHRNA1*, *CHRNA10*, *CHRNA2*, *CHRNA3*, *CHRNA4*, *CHRNA5*, *CHRNA6*, *CHRNA7*, *CHRNA9*, *CHRNB1*, *CHRNB2*, *CHRNB3*, *CHRNB4*, *CHRND*, *CHRNE,* and *CHRNG*). One gene influenced nicotine metabolism (*CYP2A6*). From the cascade of reward, two serotoninergic genes (*SLC6A4* and *TPH1*), three opioid receptors (*OPRM1*, *OPRD1,* and *OPRK1*), three GABA (*GABBR2*, *GABRA2,* and *GABRA4*) and four dopaminergic genes (*COMT*, *DDC*, *DRD2,* and *SLC6A3*) were associated with TUD. Two other genes belonging neither to the nicotine metabolism nor to the cascade theory of reward were selected (*NRXN1* and *SIGMAR1*). *NRXN1* encodes for a synaptic neuronal adhesion molecule and *SIGMAR1* is implicated in cellular differentiation, neuroplasticity, neuroprotection and cognitive functioning of the brain [11].

**Table 1:** Genes associated to tobacco use disorder selected from Gene2Mesh and Ingenuity

**Gene**	**Gene function**	**Gene2Mesh**	**Ingenuity**
*CHAT*	Nicotinic receptor	X	
*CHRNA1*	Nicotinic receptor		X
*CHRNA2*	Nicotinic receptor		X
*CHRNA3*	Nicotinic receptor	X	X
*CHRNA4*	Nicotinic receptor	X	X
*CHRNA5*	Nicotinic receptor	X	X
*CHRNA6*	Nicotinic receptor		X
*CHRNA7*	Nicotinic receptor		X
*CHRNA9*	Nicotinic receptor		X
*CHRNA10*	Nicotinic receptor		X
*CHRNB1*	Nicotinic receptor		X
*CHRNB2*	Nicotinic receptor		X
*CHRNB3*	Nicotinic receptor		X
*CHRNB4*	Nicotinic receptor		X
*CHRND*	Nicotinic receptor		X
*CHRNE*	Nicotinic receptor		X
*CHRNG*	Nicotinic receptor		X
*COMT*	Dopamine	X	
*CYP2A6*	Nicotine metabolism	X	
*DDC*	Dopamine	X	X
*DRD2*	Dopamine	X	
*GABBR2*	GABA		X
*GABRA2*	GABA	X	
*GABRA4*	GABA	X	
*NRXN1*	Neurexin		X
*OPRD1*	Opioid receptor		X
*OPRM1*	Opioid receptor		X
*OPRK1*	Opioid receptor		X
*SIGMAR1*	Non-opioid receptor		X
*SLC6A3*	Dopamine	X	X
*SLC6A4*	Serotonin	X	X
*TPH1*	Serotonin	X	

Selected genes are represented by X; GABA, Gamma-aminobutyric acid.

Figure 2 and Figure 3 show the smallest network hypothesized by IPA and STRING based on the 32 selected genes. In IPA, two groups of nicotinic receptors were connected. However, most genes, such as those influencing the cascade theory of reward, were not interconnected (Figure 2). In STRING, with a confidence level of 0.900, two groups of genes were found to be connected. The first one included mainly genes from the cascade theory of reward (*DDC*, *TPH1*, *SLC6A4*, *COMT*, *DRD2*, *OPRM1*, *OPRK1*, *OPRD1*, and *SLC6A3*) and the second included nicotinic receptors (*CHAT*, *CHRNA2*, *CHRNA3*, *CHRNA4*, *CHRNA5*, *CHRNA7*, *CHRNB2,* and *CHRNB4*) (Figure 3).

### Candidate disorder selection

Of the 20 disorders selected using the two selection methods, 14 were in common (Table 2). These disorders covered a broad range of categories including substance related disorders (addiction and alcoholism), psychiatric disorders (depressive disorders, schizophrenia, and schizoaffective disorders), cancer (oesophageal cancer, gastric cancer, and bladder cancer), cardiovascular disorders (stroke, coronary disease, vascular disorder, and malignant hypertension) and psychomotor disorders (motor dysfunction and psychomotor agitation).

**Table 2:** Disorders associated to genes influencing tobacco use disorder selected from methods 1 and 2

**Disorder**	**Method 1**	**Method 2**
Addiction	X	X
Alcoholism	X	X
Bladder cancer	X	X
Cervical cancer		X
Coronary disease	X	X
Delirium	X	
Depressive disorder	X	X
Dyskinesia	X	
Gastric cancer	X	X
Gastrointestinal tract cancer		X
Hypertension		X
Insomnia	X	
Leukaemia		X
Liver cancer		X
Malignant hypertension	X	X
Mood disorders	X	
Movement disorders	X	
Motor dysfunction	X	X
Oesophageal cancer	X	X
Pancreatic cancer		X
Psychomotor agitation	X	X
Schizophrenia	X	X
Schizoaffective disorder	X	X
Stroke	X	X
Subarachnoid haemorrhage	X	
Vascular disorder	X	X

Method 1, 20 disorders with the strongest p-value indicating the relationship with the selected genes using Ingenuity Pathway Analysis; Method 2, based on category of disorders and developed in two steps: Firstly selection of the five disorders with the strongest p-value for each category and secondly limitation to the 20 disorders with the strongest p-value; Final selected disorders are the one retrieved from both method 1 and method 2.

### Genetic similarities between TUD and selected disorders

The Jaccard distance ranged from 0.00 (bladder cancer-malignant hypertension, oesophageal cancer-bladder cancer, oesophageal cancer-malignant hypertension, and alcoholism-schizoaffective disorder) to 0.45 (addiction-bladder cancer, addiction-malignant hypertension, and addiction-oesophageal cancer). With a Jaccard distance of 0.00, four pairs of disorders were exactly similar from a genetic point of view (Table 3).

**Table 3:** Jaccard distance between pairs of disorders associated to genes influencing tobacco use disorder

**Disorder**	**Addiction**	**Alcoholism**	**Bladder cancer**	**Coronary disease**	**Depressive disorder**	**Gastric cancer**	**Malignant hypertension**	**Motor dysfunction**	**Oesophageal cancer**	**Psychomotor agitation**	**Schizoaffective disorder**	**Schizophrenia**	**Stroke**	**Tobacco used disorder**
Alcoholism	**0.138**													
Bladder cancer	0.448	0.360												
Coronary disorder	0.367	*0.269*	*0.200*											
Depressive disorder	**0.133**	**0.074**	0.407	*0.259*										
Gastric cancer	*0.345*	0.370	*0.158*	*0.304*	0.414									
Malignant hypertension	0.448	0.360	**0.000**	*0.200*	0.407	*0.158*								
Motor dysfunction	*0.310*	*0.269*	*0.200*	*0.261*	*0.321*	*0.227*	*0.200*							
Oesophageal cancer	0.448	0.360	**0.000**	*0.200*	0.407	*0.158*	**0.000**	*0.200*						
Psychomotor agitation	0.379	*0.280*	**0.111**	*0.190*	*0.333*	*0.238*	**0.111**	**0.100**	**0.111**					
Schizoaffective disorder	**0.138**	**0.000**	0.360	*0.269*	**0.074**	0.370	0.360	*0.269*	0.360	*0.280*				
Schizophrenia	*0.161*	**0.107**	0.429	*0.286*	**0.103**	0.433	0.429	*0.345*	0.429	0.357	**0.107**			
Stroke	*0.241*	*0.192*	*0.273*	*0.174*	*0.250*	*0.292*	*0.273*	0.091	*0.272*	*0.182*	*0.192*	*0.276*		
Tobacco use disorder	**0.103**	*0.241*	0.384	0.413	*0.233*	*0.269*	0.385	*0.296*	0.384	0.370	*0.241*	*0.258*	0.345	
Vascular disorder	*0.258*	*0.214*	0.360	*0.200*	*0.207*	0.370	0.360	*0.200*	0.360	*0.280*	*0.214*	*0.172*	**0.120**	0.355

Colours representing the genetic similarities between disorders: bold = strong similarities (<0.15); italic = middle similarities (0.15-0.35); normal = low similarities (>0.35).

**Figure 4:**
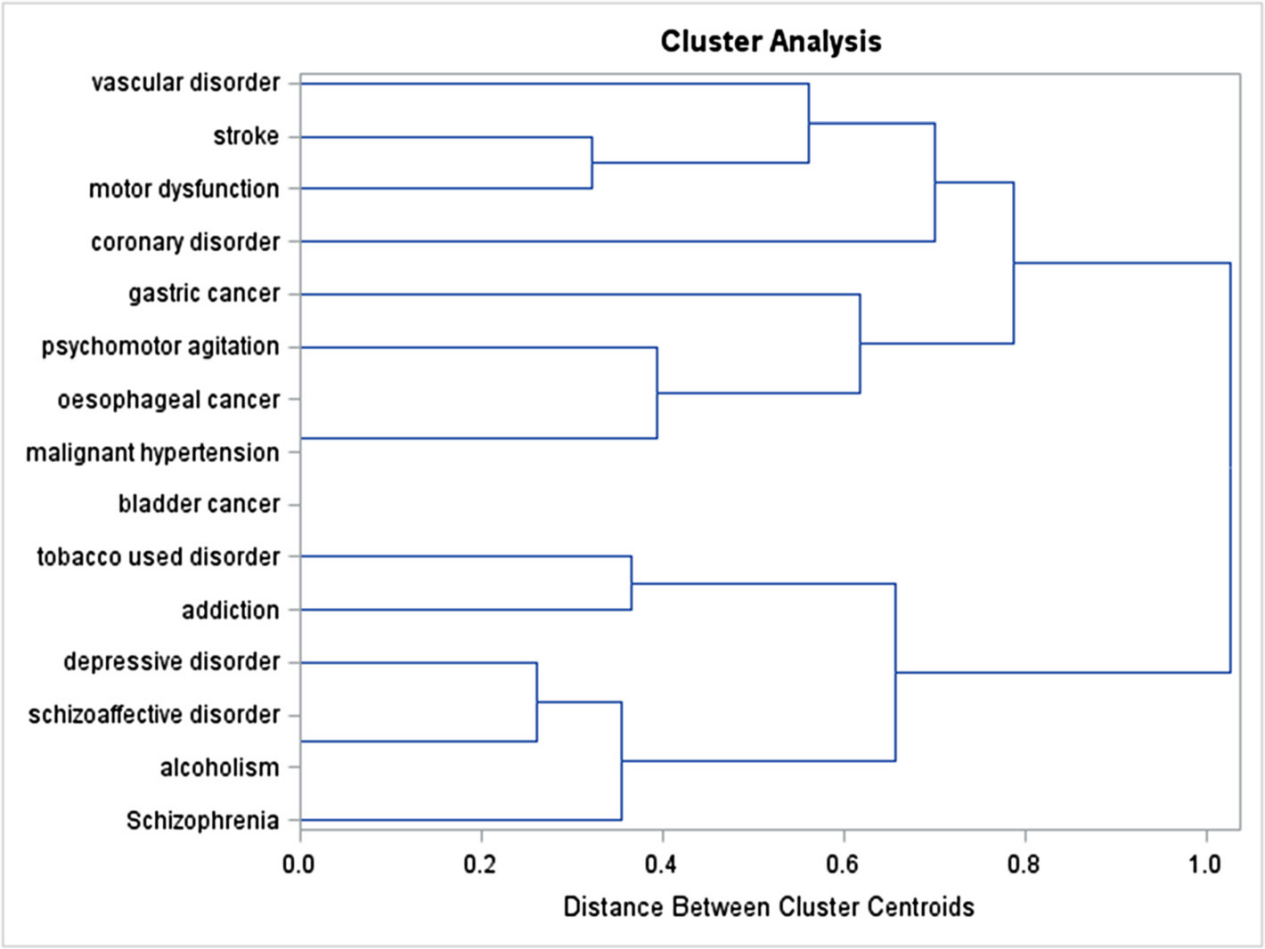
Dendrogram of disorders associated to the same genes as tobacco use disorder resulting from centroid hierarchical cluster analysis, using the Jaccard distance obtained from 32 genes.

The dendrogram obtained from the cluster analysis (Figure 4) showed two main clusters of disorders. The first cluster encompassed disorders that result from smoking (e.g. vascular disorder, gastric cancer, and malignant hypertension) [12], and the second cluster included disorders that coexist with smoking (e.g. addiction, alcoholism, and depressive disorders) [13].

## Discussion

The current exploratory study is a first step towards evaluating the genetic similarities between TUD and disorders genetically associated with TUD. Genes influencing TUD were associated with a variety of other disorders. Cluster analyses reported two main clusters of disorders. The first cluster included disorders that result from smoking (including e.g. vascular disorder, gastric cancer, and malignant hypertension) while the second cluster included disorders that coexist with smoking (including e.g. addiction, alcoholism, and depressive disorder).

To our knowledge, this is the first study to show that a literature review combined with a gene pathway analysis can provide relevant information to better understand the genetic similarities between TUD and related comorbidities. This is an important endeavour since even though genes are not the only factor linking TUD to other disorders, they are part of the mechanism leading to the development of a disorder. The relation between TUD and other disorders is also mediated by other factors including treatments, environmental aspects, and epigenomic modifications [2].

McEachin *et al.* developed a comparable analysis focusing on the association between TUD and bipolar disorder. Their genetic network analyses reported an over-representation of genes associated with those two disorders. Their gene selection, developed in Gene2Mesh, included *COMT*, *SLC6A3,* and *SLC6A4*[14]. Those three genes also appeared in the gene selection of the current study.

The observed genes mainly influenced either the cascade theory of reward or the nicotine metabolism pathways. This was not unexpected as the starting point was TUD, which is linked to the cascade theory of rewards and the nicotine metabolism pathways. Most of the selected genes were nicotinic receptors. These receptors are activated by nicotine and a prolonged contact with nicotine will lead to receptor desensitization [15]. Moreover, except *CHAT*, all nicotinic receptors influenced all selected disorders. There were only two genes that belong to neither of these groups (*SIGMAR1* and *NRXN1*). *SIGMAR1* is a protein receptor involved in the modulation of glutamatergic and dopaminergic neurotransmission [11,16]. *NRXN1* is a presynaptic neuronal adhesion molecule. However, its biological association with multiple disorders (e.g. autism and schizophrenia) is still unclear [11,17].The reason why the networks developed in IPA and STRING (Figure 2) were different might be due to the selection criteria. Indeed, the options in IPA were very stringent, whereas STRING used a confidence level of 0.90. STRING utilises pathway and physical interaction databases, including BIND, KEGG, and bioGrid. STRING infers links between proteins from genomic associations using its own unique scoring method. It employs automated parsing of scientific texts from SGD, OMIM, FlyBase and PubMed. IPA uses the QIAGEN Ingenuity Knowledge Base which is updated with automated text mining, which is then manually curated, literature findings from PhD level experts, manual curation of pathways by in-house experts, and a wide variety of third-party databases, including major NCBI databases. Ingenuity uses a wider range of third party databases, which also include clinical biomarker databases, gene expression databases, and metabolomic databases. The differences in third-party databases used by STRING and IPA, differences in the proprietary text-mining algorithms used to search the literature, and differences in manual curation by the experts employed by STRING and IPA may influence the outcome.

All expected categories of disorders associated with TUD were represented in our disease selection. Due to the limitation in the number of selected disorders, and to enhance the feasibility of the analysis, some specific disorders associated with TUD were not retrieved through our methods of selection. This is, for example, the case for lung cancer [12], attention deficit disorder with hyperactivity (ADHD) [18], and bipolar disorder [14].

Moreover, in the literature, not only links between (i) TUD and mental illness, (ii) TUD and cardiovascular disease, or (iii) TUD and cancer were observed. For example, depression has been reported to increase the risk of cardiovascular disease. The reason of this association may be the non-compliance to treatment, shared risk factors in the two disorders (e.g. smoking and hypertension), or physiologic factors (e.g. the activation of the hypothalamic-pituitary-adrenocortical axis and inflammation) [19].

The prevalence of disorders that coexists with smoking including mental health and addictive disorders appears to be 2 to 4 times higher in smokers than in the general population. Shared genetic variation of *CHRNA7* is, for example, reported in TUD and schizophrenia [20]. Regarding disorders that result from smoking, the most plausible theory is epigenomic changes due to carcinogenic compounds of tobacco [21]. For example, it was reported that smoking induced a down-regulation of the interferon *IFIT1* involved in the progression and invasiveness of bladder cancer [22]. Therefore, genes are just one of the pathways linking TUD and related disorders.

Psychiatric disorders are influenced by multiple factors and it is conceivable that genes influence the biological mechanisms that underlie the psychopathology. They have, for example, an impact on the association between dorsolateral prefrontal cortex and hippocampus or striatum. This hypothesis is supported by the following example; *COMT* has been consistently associated with prefrontal regulation of dopamine, which indicates a risk of schizophrenia due to the reduced signal in the prefrontal cortex [23]. However, inconsistent results were obtained when studying the direct link between *COMT* and schizophrenia [23]. In TUD, intermediate phenotypes may also be used to better understand the link with other disorders. For disorders that result from smoking, one plausible intermediate phenotype may be tobacco smoking as smoking is a main factor to develop those disorders. Moreover, reward deficiency syndrome (RDS) is probably an important intermediate phenotype of disorders that coexist with smoking. RDS is a hypodopaminergic state including 4 main types of behavioural disorders: addictive behaviour (e.g. substance abuse or obesity), impulsive behaviour (e.g. attention deficit hyperactivity disorder or Tourette syndrome), compulsive behaviour (e.g. aberrant sexual behaviour or pathological gambling), and personality disorder (e.g. conduct disorder or aggressive disorder) [24]. Among others, the following variants influencing the dopamine pathway were suggested to induce RDS: *DRD2* TaqIA A1, *DRD2* 957 T, *SLC6A3* VNTR 9R, and *COMT* Val158Met ValVal [24,25]. This enhanced the need of individualized interventions in case of, for example, smoking cessation. Indeed, if a smoker with depressive disorder used cigarettes as a self-medication to enhance the release of dopamine, then, during smoking cessation the treatment of depression will need adaptation [18].

Previous publications such as Carlsten *et al.* already pointed out the benefits of personalized medicine in TUD [26]. Due to the strong evidence that genetic factors are involved in nicotine addiction and smoking cessation, interventions should be adapted in terms of the type of treatment, the dose, and the duration. For example, bupropion that modifies the activity of dopamine may have an impact on smoking relapse [27]. This may partly explain why individuals respond better to one treatment than another [28]. Therefore, as proposed by Walton *et al.*, there is a need to improve the ‘understanding of the molecular mechanisms that underlie tobacco addiction’ [28]. This understanding of the molecular mechanisms may also include those linking TUD to other disorders.

### Limitations

Due to the novelty of the methods used, the current study may be criticized and could be improved in the near future with the development of a new software and improvement of the methodology.

Regarding the search strategy, the most important limitation was the low number of publications focusing on European populations. Among other studies, there was a relatively high number of publications focusing on Asian populations or on mixed ethnicities. Based on that observation, other genes may also be associated with TUD. The aim of the current study was not to be exhaustive in the gene and disorder selection, but was rather exploratory.

The use of Gene2MeSH allowed screening of publications on PubMed in an easier way compared with screening directly through PubMed and helped in a more systematic and comprehensive screening of the literature. Looking into other databases may have increased the number of selected genes and disorders and enhanced the number of relations between selected genes and disorders. The additional use of IPA in the gene and disease selection enhanced our review as it includes text-mining research and various databases. Nevertheless, in IPA there was no possibility to limit the search based on specific inclusion and exclusion criteria, such as those chosen in the literature review (focusing on populations of European ancestries, and excluding studies with whole population having a specific disorder or trait). Therefore, some associations retrieved from IPA may not be specific to our inclusion criteria. Focusing on genes associated with TUD may enrich the results with disorders that were directly influenced by smoking. Eliminating associations described in only one publication and careful review of the found literature may help reduce potential confounding, although elimination of confounding may be difficult as the object was to find disorders that use similar pathways as TUD.Networks based on literature-mining, as developed in IPA and STRING (Figures 2 and 3), may introduce false positivity because it did not take into account the true biological relation between the elements.

The literature used contained data from Single Nucleotide Polymorphism (SNP; genetic) studies and expression (genomic) studies. It is well known that SNPs can influence expression and or function of a protein. Unfortunately, it is not known for all SNPs whether they influence expression and/or function of a protein, nor does expression data give insight into the functionality of a protein (i.e. high expression of a non-functional (mutated) protein has a completely different effect compared to a functional protein). These uncertainties can alter the interactions in the pathway under construction and can be limited by careful manual curation of the search results during the candidate gene selection.

Further studies might take epigenomics into account to gain insight from the dynamics of the gene-environment interactions and comorbidities. Exposomes might be linked to health effects even if to date no direct association has been reported [29]. However, genetics, epigenomics, and exposome are just part of the whole pattern explaining the relation between TUD and other disorders. This explains the interindividual differences.

## Conclusions

This exploratory study may partially explain the genetic similarities between TUD and disorders using the same genetic pathways. A better understanding of the disorders linked to the same genes as TUD may contribute in individualizing and personalizing care related to TUD. Indeed, developing a holistic approach to treat TUD by taking into account the different factors that may influence a trait or a disorder, has been suggested as the best practice to improve health.

In the future, the type of analyses that were developed in this research might be extended to individual characteristics, the effects of treatments, or the severity of symptoms. Moreover, the mechanisms linking TUD to disorders might be assessed in twin or prospective studies. This may give insights into whether the relation is due to causality or pleiotropy. Finally, analysis of micro-array online databases for the most significant diseases may give an idea whether the expression is comparable between disorders.

## Competing interests

The authors declare that they have no competing interests.

## Authors’ contribution

SDV participated in the data collection, performed the statistical analyses and drafted the manuscript. SO and SAM participated and supervised the data collection. SO, SAM, AB and HVO helped to draft the manuscript. All authors read and approved the final manuscript.

## Pre-publication history

The pre-publication history for this paper can be accessed here:

http://www.biomedcentral.com/1471-2350/15/85/prepub
